# Engineering Blood and Lymphatic Microvascular Networks in Fibrin Matrices

**DOI:** 10.3389/fbioe.2017.00025

**Published:** 2017-04-18

**Authors:** Lea Knezevic, Mira Schaupper, Severin Mühleder, Katharina Schimek, Tobias Hasenberg, Uwe Marx, Eleni Priglinger, Heinz Redl, Wolfgang Holnthoner

**Affiliations:** ^1^Ludwig Boltzmann Institute for Experimental and Clinical Traumatology, Vienna, Austria; ^2^Austrian Cluster for Tissue Regeneration, Vienna, Austria; ^3^Department of Cardiology, Division of Heart and Lungs, University Medical Center Utrecht, Utrecht, Netherlands; ^4^Technische Universität Berlin, Medical Biotechnology, Berlin, Germany; ^5^TissUse GmbH, Berlin, Germany

**Keywords:** lymphatics, endothelial cells, coculture, fibrin, tissue engineering

## Abstract

Vascular network engineering is essential for nutrient delivery to tissue-engineered constructs and, consequently, their survival. In addition, the functionality of tissues also depends on tissue drainage and immune cell accessibility, which are the main functions of the lymphatic system. Engineering both the blood and lymphatic microvasculature would advance the survival and functionality of tissue-engineered constructs. The aim of this study was to isolate pure populations of lymphatic endothelial cells (LEC) and blood vascular endothelial cells (BEC) from human dermal microvascular endothelial cells and to study their network formation in our previously described coculture model with adipose-derived stromal cells (ASC) in fibrin scaffolds. We could follow the network development over a period of 4 weeks by fluorescently labeling the cells. We show that LEC and BEC form separate networks, which are morphologically distinguishable and sustainable over several weeks. In addition, lymphatic network development was dependent on vascular endothelial growth factor (VEGF)-C, resulting in denser networks with increasing VEGF-C concentration. Finally, we confirm the necessity of cell–cell contact between endothelial cells and ASC for the formation of both blood and lymphatic microvascular networks. This model represents a valuable platform for *in vitro* drug testing and for the future *in vivo* studies on lymphatic and blood microvascularization.

## Introduction

Survival of tissue-engineered constructs following implantation is inherently dependent on adequate oxygen and nutrient supply. The diffusion range of oxygen is generally limited to ~200 μm (Carmeliet and Jain, 2000), meaning that thick tissues require a vascular network to deliver a sufficient amount of nutrients and, thus, promote cell survival (Frerich et al., 2001; Costa-Almeida et al., 2014). Consequently, adequate oxygen and nutrient supply is a prerequisite for survival of tissue-engineered constructs *in vivo*. Therefore, the success of scaffold inclusion relies greatly on efficient vascularization. While a lot of attention has been dedicated to the engineering of blood vasculature, substantially fewer studies have focused on the engineering of lymphatic vessels. Lymphatic research gained a boost with the identification of markers that distinguish blood and lymphatic endothelial cells (LEC) [blood vascular endothelial cells (BEC) and LEC, respectively]. The lymphatic system is an essential component of nearly all tissues; its main functions being drainage and recycling of interstitial fluid, and immune cell and lipid transport (Tammela and Alitalo, 2010). Tissues with damaged lymphatic networks are characterized by lymphedema and persistent infections (Petrek et al., 2001; Beesley et al., 2007; Hayes et al., 2008; Weitman et al., 2013). Consequently, incorporation of a lymphatic network into tissue-engineered constructs is likely to improve their integration and functionality *in vivo* by enabling appropriate tissue drainage and substantial immune cell accessibility.

A large body of research has focused on overcoming the issue of insufficient vascularization (Rouwkema et al., 2008; Baldwin et al., 2014). Pre-vascularization strategies assume the development of a vascular network *in vitro* prior to implantation of the tissue-engineered construct. Coculturing endothelial cells together with supporting cells was shown to result in the formation of a vascular network *in vitro* (Rivron et al., 2008; Baldwin et al., 2014; Costa-Almeida et al., 2014). Furthermore, supporting cells are needed for stabilization of the vessels in networks, and for the restriction of permeability and prevention of vessel regression (Kunz-Schughart et al., 2006; Rivron et al., 2008; Duttenhoefer et al., 2013). The supporting cells most commonly used include fibroblasts (Grainger and Putnam, 2011), mesenchymal stromal cells, such as adipose-derived stromal cells (ASC) and bone marrow-derived stromal cells [reviewed by Pill et al. (2015)], and smooth muscle cells (Elbjeirami and West, 2006). A source of endothelial cells commonly used is human umbilical vein endothelial cells (HUVEC) (Siow, 2012), which have been shown to form vascular networks when cocultured with supporting cell types *in vitro* (Sorrell et al., 2007; Verseijden et al., 2010a; Holnthoner et al., 2015). On the other hand, human dermal microvascular endothelial cells (HDMEC) represent a more appealing source, since the majority of endothelial cells in a human body are found in microvascular structures, making this cell type suitable for studying many physiological and pathological conditions (Hewett and Murray, 1993). These cells have also been found to form vascular networks when cocultured with mural cells (Sorrell et al., 2007; Unger et al., 2010), but their behavior resembles that of *in vivo* cells more closely than it does of HUVEC (Swerlick et al., 1991; Cornelius et al., 1995). In addition to being a valuable source of BEC, HDMEC also contain LEC (Kriehuber et al., 2001; Marino et al., 2014; Gibot et al., 2016). For a potential clinical application, circulating endothelial colony-forming cells (ECFC) are a promising source of BEC, since they can be isolated from peripheral blood (Siow, 2012) and show tube-forming capacity and the ability to form vascular networks *in vitro* (Fuchs et al., 2009; Medina et al., 2010; Holnthoner et al., 2015). Moreover, they have been shown to contain a subset of lymphatic ECFC (DiMaio et al., 2016) and could also offer the possibility of engineering an autologous vascular network for future clinical use.

A few approaches for lymphatic tissue engineering have been developed in response to the necessity for tissue drainage and immune cell surveillance of tissue constructs (Schaupper et al., 2016). Lymphatic capillary-like structures, for example, were established in collagen and fibrin matrices with interstitial flow applied (Helm et al., 2007). Similarly, lymphatic capillary formation was achieved in a skin regeneration model implanted into mouse tails, simulating the influence of interstitial flow (Boardman and Swartz, 2003). Lymphatic capillaries were successfully formed in fibrin-collagen hydrogels and integrated into a rat model of dermo-epidermal skin grafts (Marino et al., 2014). Another model shows the development of lymphatic capillaries in an *in vitro* scaffold-free three-dimensional (3D) coculture of LEC and fibroblasts (Gibot et al., 2016).

The functionality of tissue-engineered constructs could be greatly improved by the simultaneous engineering of a lymphatic vascular network as well as a blood vascular network. The aim of this study was to investigate the network-forming capacities of the two endothelial cell populations isolated from the HDMEC population and to explore the differences between the two cell types in vascular network formation *in vitro*. Consequently, we have established a 3D coculture model consisting of ASC and fluorescently labeled LEC and BEC in fibrin hydrogels and showed the effect of vascular endothelial growth factor (VEGF)-C and supporting cells on the development of blood and lymphatic networks.

## Materials and Methods

### Cell Culture and Isolation

The isolation of human ASC was approved by the ethics committee of the state of Upper Austria (#200). ASC were obtained from liposuction materials as described previously (Wolbank et al., 2007) and cultured in Endothelial Cell Growth Medium (EGM™-2 BulletKit™, Lonza) supplemented with additional FCS (Sigma-Aldrich, St. Louis, MO, USA) to a final concentration of 5% (referred to as EGM-2 unless stated otherwise). The cells were used in passages 2–10. HDMEC were isolated from human foreskin, according to protocol described previously (Schimek et al., 2013). Juvenile prepuce was obtained in compliance with the relevant laws, with informed consent and ethics approval (Ethic Committee Charité University Medicine, Berlin, Germany), from a pediatric surgery after routine circumcisions. LEC were isolated from HDMEC populations using immunomagnetic cell sorting with anti-podoplanin antibody and magnetic beads (Dynabeads, M280 goat anti-rabbit, ThermoFisher), following the manufacturer’s instructions. The podoplanin-negative population, representing BEC, were obtained from the negative fraction by a second negative immunomagnetic selection with anti-podoplanin antibody, to remove the remaining podoplanin positive cells. BEC were cultured in EGM-2 medium, while LEC were propagated in EGM-2 medium supplemented with 50 ng/mL VEGF-C. All cells derived from HDMEC were used between passages 6 and 9.

### Plasmids and Retroviral Infection

Isolated LEC and BEC populations were retrovirally infected with fluorescent proteins to visualize network formation. The cDNA encoding pLV-EGFP, pLV-mCherry, and pBMN-Z was purchased from Addgene (Cambridge, MA, USA). The pcDNA3-EYFP-HIS was purchased from Invitrogen (ThermoFisher, Waltham, MA, USA). The eGFP and mCherry were subcloned into the pBMN backbone after digestion with *Bam*HI and *Sal*I. The EYFP-HIS was subcloned into pBMN after digestion with *Bam*HI and *Eco*RI. Phoenix Ampho cells were a kind gift from Regina Grillari (University of Natural Resources and Life Sciences, Vienna, Austria) and cultured in DMEM 10% FCS. Virus particle generation was performed by transfecting Phoenix cells at 80% confluency using Lipofectamine 2000 or TurboFect (ThermoFisher, Waltham, MA, USA), following the manufacturer’s instructions. The supernatants containing virus particles were transferred onto 80% confluent LEC and BEC, and virus particles were centrifuged onto the cells at 800 × *g* for 60 min. The supernatant was then removed, and the cells were incubated overnight in EGM-2. Retrovirally infected cells were then expanded in new flasks and used for subsequent experiments.

### Two-dimensional (2D) Cocultures

Two-dimensional cocultures were prepared in 8-well chamber slides by seeding a total number of 48,000 cells/well. The cultures prepared contained equal ratios of ASC, LEC, and BEC, cultured in EGM-2 medium, with or without VEGF-C (25 ng/mL).

### Embedding of the Cells in Fibrin Matrices

Fibrin matrix was prepared, as described elsewhere (Rohringer et al., 2014), to address the question whether lymphatic capillaries can be built within the fibrin hydrogel. LEC and BEC used in these cultures were fluorescently labeled as described above. Briefly, 3D matrices were prepared on 15-mm diameter round coverslips (Paul Marienfeld, Lauda Königshofen, Germany) and are subsequently referred to as “clots.” The clots were prepared by diluting thrombin (human thrombin 4, Baxter, Vienna, Austria; 4 U/mL) 1:10 in calcium chloride (CaCl_2_, Baxter, Vienna, Austria or Fresenius Kabi GmbH, Graz, Austria; 40 µmol/mL solution), and fibrinogen (Baxter, Vienna, Austria; 100 mg/mL) 1:20 in EGM-2. A total of 100,000 cells/cell type were used in each clot. The clot suspension contained a final concentration of 0.2 U/mL thrombin and 2.5 mg/mL fibrinogen. All clots were prepared in duplicate. After placing the suspensions on the cover slides, the clots were incubated for 20 min at 37°C prior to the addition of medium. An amount of 2 mL of EGM-2 medium or EGM-2 medium supplemented with VEGF-C at indicated concentrations was added to the clots and exchanged every 2–3 days.

### LEC and BEC 3D Cocultures with Varying VEGF-C

Either LEC or BEC were cocultured with ASC and placed into fibrin matrices, as indicated above. Clots were cultured in EGM-2 with different VEGF-C concentrations (0, 10, 25, and 50 ng/mL). Fluorescence images of the cultures were taken once a week for 4 weeks.

### LEC and BEC 3D Tricultures

Lymphatic endothelial cells and BEC were embedded in fibrin matrices, as indicated above and cultured separately (with or without ASC) or together (with or without ASC) in EGM-2 or, for LEC, in EGM-2 medium containing VEGF-C (25 ng/mL). The cultures were photographed once a week for 4 weeks.

### Generation of ASC-Conditioned Medium (ASC-CM) and 3D Coculture

A total of 100,000 ASC were seeded in a tissue culture flask with the surface area of 75 cm^2^. The cells were cultured for 48 h prior to the removal of supernatant. The supernatant was removed and centrifuged for 15 min at 500 × *g*. The cells were then split and cultured for the next supernatant generation. As a control, EGM-2 medium was incubated in a tissue culture flask for the same time period as the ASC-CM. The 3D fibrin matrices were prepared as indicated earlier. LEC and BEC were cultured alone or with ASC. When cultured alone, the cells were either incubated in control EGM-2 medium or with ASC-conditioned EGM-2 medium (2 mL/clot). When cultured with ASC, the cells were cultured in a 1:1 ratio (100,000 cells of each) and grown in EGM-2 medium. When indicated, the medium was supplemented with VEGF-C at a concentration of 25 ng/mL. Images of the cultures were taken once a week.

### Network Quantification

The images were first processed in ImageJ software. The networks were quantified using Adobe Photoshop (Adobe Systems, San Jose, CA, USA) and Angiosys (Buckingham, UK) software. The standardization of images was performed in Adobe Photoshop, as described previously (Charwat et al., 2015). Briefly, the images of the LEC and BEC 3D tricultures were taken after 7 days from four different positions in each clot. One image was taken from each sample (including the duplicates) for three separate experiments (with cells from three donors) at day 28 for the quantification of the LEC networks at different VEGF-C concentrations.

### Antibodies and Flow Cytometry

Antibodies used in flow cytometry measurements were as follows: anti-rabbit PE (Poly4064) and anti-mouse PE (Poly4053) from BioLegend (San Diego, CA, USA); mouse antihuman podoplanin from Santa Cruz Biotechnology (Dallas, TX, USA); rabbit antihuman podoplanin, rabbit antihuman LYVE-1, and mouse antihuman vascular endothelial growth factor receptor 3 (VEGFR3) from Reliatech (Wolfenbüttel, Germany); and anti-rabbit and anti-mouse Alexa Fluor 488 from ThermoFisher Scientific (Waltham, MA, USA). The analysis was performed on a Beckman Coulter CytoFlex Flow Cytometer (Brea, CA, USA), and the data were analyzed using FlowJo software (FlowJo LLC, Ashland, OR, USA).

### Statistical Analysis

The statistical analysis for LEC and BEC cocultures was performed with GraphPad software using the Student’s *t*-test, with a level of significance set at *p* < 0.05. The data were logarithmically transformed and statistical analysis performed using one-way ANOVA with Dunnett’s *post hoc* analysis for the quantification of the networks at different VEGF-C concentrations. Level of significance was set at *p* < 0.05.

## Results

### Isolation and Characterization of LEC and BEC from HDMEC Cultures

**Figure 1:**
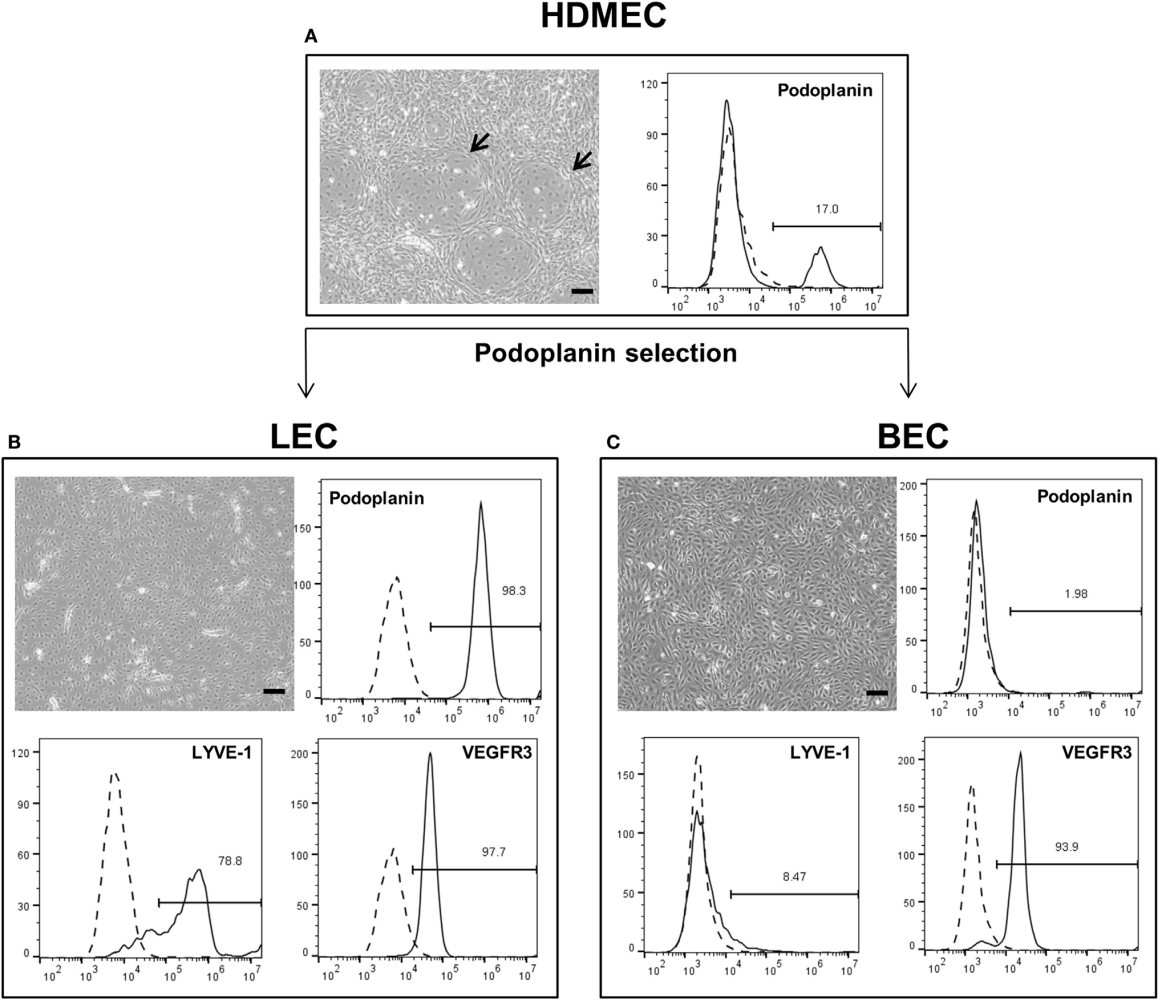
**Schematic representation of the separation of human dermal microvascular endothelial cells (HDMEC) into lymphatic endothelial cells (LEC) and blood vascular endothelial cells (BEC)**. Scale bar represents 100 µm. **(A)** Brightfield microscopy image of HDMEC before MACS with representative FACS plot showing the presence of podoplanin-positive cells. Arrows indicate LEC islands within BEC populations. **(B)** Brightfield microcopy images and representative FACS plots of a pure LEC population. The population is positive for lymphatic markers podoplanin, LYVE-1, and vascular endothelial growth factor receptor 3 (VEGFR3). **(C)** Brightfield microcopy images and representative FACS plots of a pure BEC population. The population is largely negative for lymphatic markers podoplanin and LYVE-1, but positive for VEGFR3.

Lymphatic endothelial cells and BEC were firstly isolated from mixed populations of HDMEC obtained from neonatal foreskins of three donors with the aim of obtaining both LEC and BEC from the same tissue source. The populations of LEC and BEC could be distinguished upon microscopic observation, where LEC are visible as island-forming cells within BEC populations (Figure 1A). In addition, the two cell types could be distinguished by the presence or absence of podoplanin (Figure 1A). After immunomagnetic cell sorting into podoplanin-positive (LEC) (Figure 1B) and podoplanin-negative (BEC) cells (Figure 1C), the purity of the populations was confirmed with flow cytometry showing >96% podoplanin-positive and -negative populations, respectively. In addition, BEC and LEC could be distinguished by lymphatic vessel endothelial hyaluronic acid receptor (LYVE)-1 expression, while both populations expressed VEGFR3 (Figures 1B,C). Populations of LEC and BEC were transduced with retroviral vectors for stable expression of GFP, YFP, or mCherry with the aim of tracking network development over several weeks.

### 2D Cultures of LEC, BEC, and ASC Show Separate LEC and BEC Vascular-Like Structures

**Figure 2:**
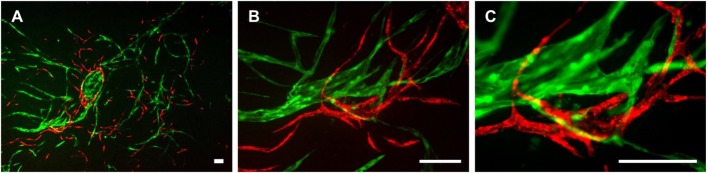
**Two-dimensional culture of lymphatic endothelial cells (LEC) (green), blood vascular endothelial cells (BEC) (red), and adipose-derived stromal cells (non-fluorescent)**. Scale bar represents 200 µm. **(A)** An overview of the culture showing elongated structures formed by LEC and BEC, and an LEC island surrounded by BEC. **(B)** The same image in larger magnification depicting the different morphologies of BEC and LEC structures, with BEC forming thin, uniform projections, and LEC forming both wide and thin structures. **(C)** The same image in higher magnification showing that structures formed by BEC and LEC are separate from each other. LEC were transduced with GFP and BEC with mCherry. LEC and BEC originate from the same human dermal microvascular endothelial cells (HDMEC) donor in a single experiment. Images are representative of three experiments with three HDMEC donors.

The three cell types were mixed in equal ratios and observed for a period of 1 week to analyze whether LEC and BEC form vascular-like structures when cocultured with ASC. The cells organized into islands of LEC (green), surrounded by BEC (red) (Figure 2A). Both LEC and BEC formed elongated tube-like structures that differed in morphology, with LEC forming wide structures and BEC thinner ones (Figures 2B,C). In addition, the LEC and BEC structures appeared separate from one another.

### LEC Respond to Increased VEGF-C Concentrations with Increased Network Densities

**Figure 3:**
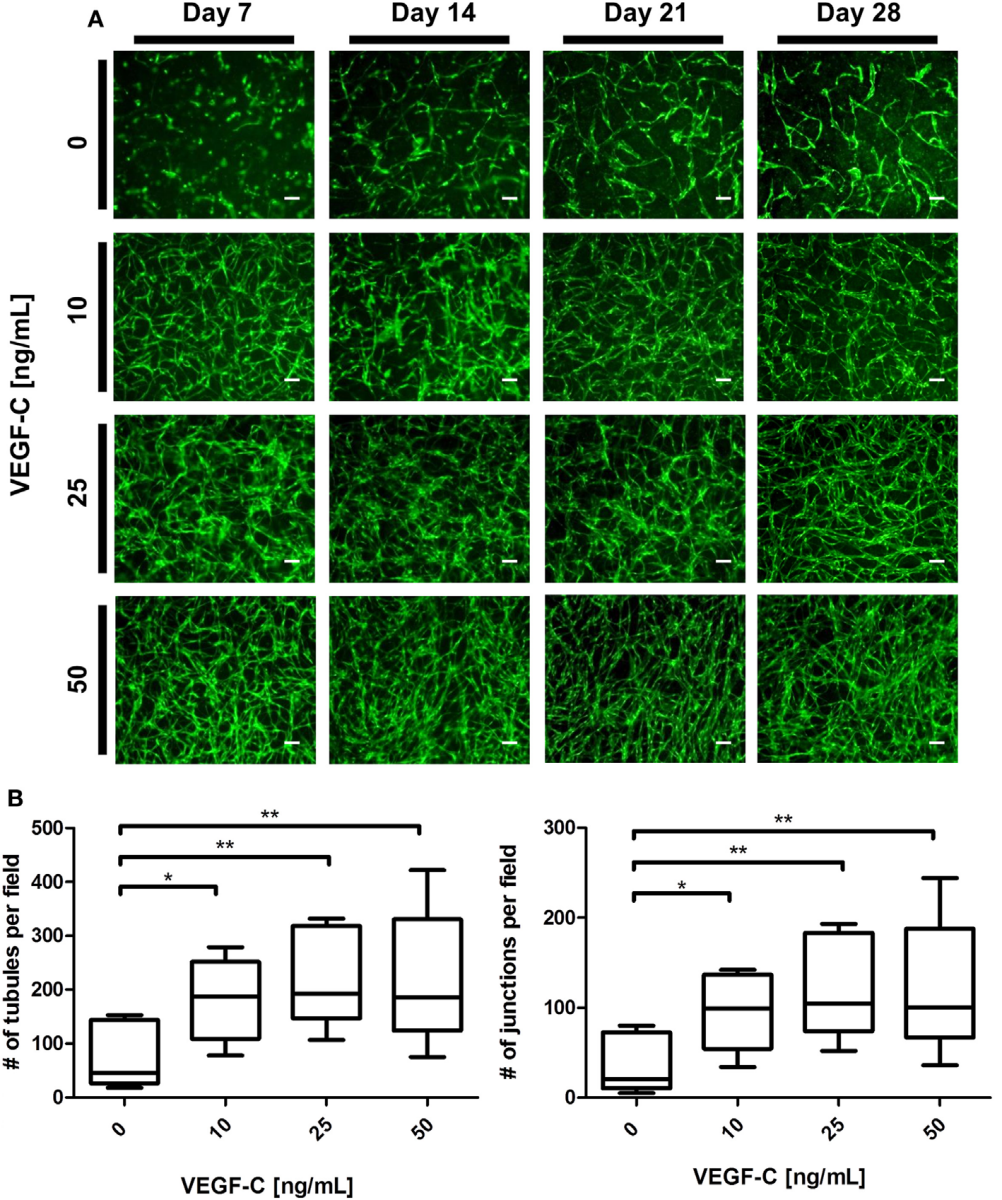
**Effect of increasing vascular endothelial growth factor (VEGF)-C concentration on lymphatic endothelial cells (LEC) (green) network formation in fibrin matrix, when cocultured with adipose-derived stromal cells (ASC) for 4 weeks**. Scale bar represents 100 µm. **(A)** Fluorescent images of LEC coculture with ASC over a period of 4 weeks, showing that LEC form more extensive networks with the increasing VEGF-C concentration. Images are representative of the results of three separate experiments (with three different LEC donors). LEC were transduced with YFP. **(B)** Quantification of networks originating from three different LEC donors, with images of two clots per donor (**p < 0.02, *p < 0.05). Statistical analysis was performed using one-way ANOVA and Dunnett’s *post hoc* test on logarithmically transformed data. All conditions per LEC donor were prepared in duplicate, and one field image was taken from each clot and corresponding duplicate.

We next aimed to determine whether LEC cocultured with ASC can form networks in fibrin hydrogels. LEC were integrated into fibrin matrices together with ASC and cultured for the period of 28 days. Due to previous observations of our group that VEGF-C is not secreted by ASC (Rohringer et al., 2014), different concentrations of VEGF-C were added to the culture medium to determine the optimal concentration of VEGF-C for network development of LEC. LEC did not form network-like structures without any exogenously added VEGF-C (Figure 3A). Some of the structures formed were elongated and resembled primitive LEC tubes, but the structures were not interconnected. An extensive network was already present at day 7 with 10 ng/mL, and the vessel-like structures were easily distinguishable. With concentrations of 25 and 50 ng/mL, the network was dense from day 7, with only subtle differences in network density between the two conditions. In addition, we observed that the structure morphology is dependent on the VEGF-C concentration. At a concentration of 10 and 25 ng/mL, the network consists of wider vessel-like structures, while at 50 ng/mL, the network contains thinner structures and these are already present at day 7. The statistical analysis shows that the concentration of 10 ng/mL increases the number of tubes and junctions significantly compared to the control condition of no exogenously added VEGF-C, while this effect was even further enhanced at concentrations of 25 and 50 ng/mL (Figure 3B). The mean number of tubules and junctions at 0 ng/mL was 72 (±59.22) and 35 (±31.97), at 10 ng/mL was 182 (±79.92) and 95 (±44.65), at 25 ng/mL was 217 (±88.98) and 119 (±55.97), and at 50 ng/mL was 218 (±125.9) and 121 (±75.31), respectively. In contrast to LEC, culturing BEC with increasing concentrations of VEGF-C did not enhance network formation (data not shown).

### Tricultures of LEC, BEC, and ASC in Fibrin Gels Show VEGF-C-Dependent Separate Network Formation of LEC and BEC

**Figure 4:**
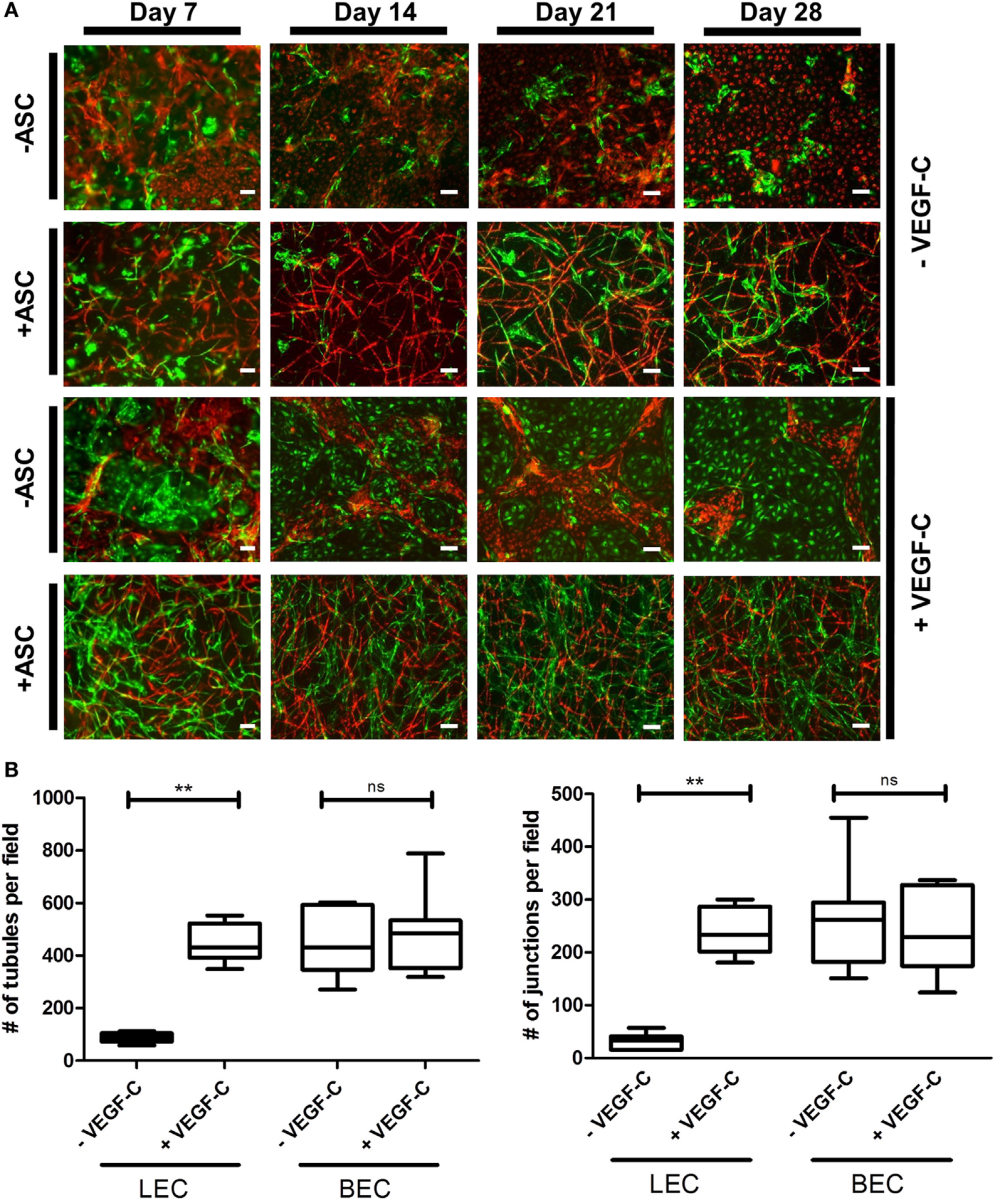
**Network formation and quantification of coculture of lymphatic endothelial cells (LEC) (green) and blood vascular endothelial cells (BEC) (red) in fibrin matrices when cultured in the presence or absence of adipose-derived stromal cells (ASC), and exogenous vascular endothelial growth factor (VEGF)-C (25 ng/mL), during the period of 4 weeks**. Scale bar represents 100 µm. **(A)** Overlays of fluorescent images of cocultures show the network formation of LEC and BEC in the presence of ASC. The addition of exogenous VEGF-C results in a denser LEC, but not BEC networks. There is no network formation of LEC or BEC without ASC. Images are representative of data from two experiments with two separate human dermal microvascular endothelial cells (HDMEC) and ASC donors. The LEC were transduced with GFP and BEC with mCherry. **(B)** Network quantification data show that there is a significant increase in the number of tubules and junctions of LEC cultured with ASC when the medium was supplemented with VEGF-C (25 ng/mL), compared to when cultured in EGM-2 medium without VEGF-C. BEC do not show a significant difference in the number of junctions or tubules between the two conditions. **p < 0.02, LEC cultured in EGM-2 vs. in EGM-2 + 25 ng/mL VEGF-C; BEC cultured in EGM-2 vs. in EGM-2 + 25 ng/mL VEGF-C. Data originate from two experiments with two HDMEC and two ASC donors.

**Figure 5:**
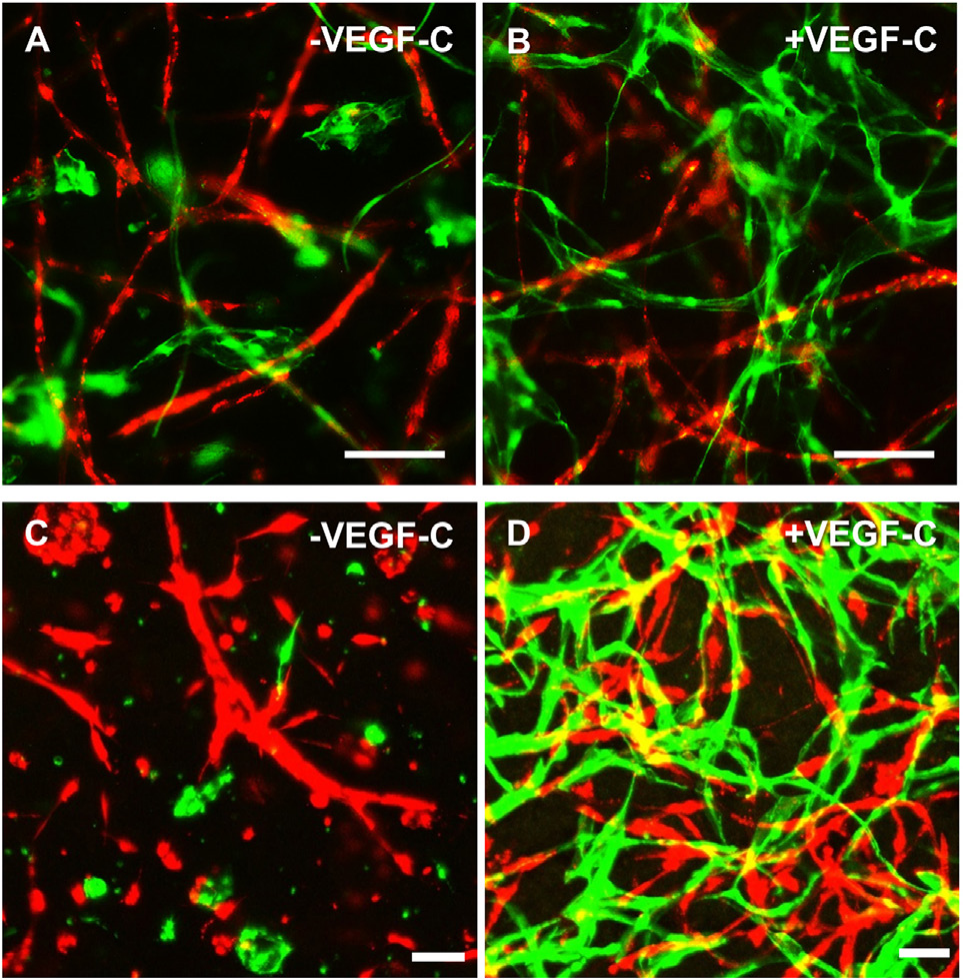
**Lymphatic endothelial cells (LEC) (green) and blood vascular endothelial cells (BEC) (red) form two separate networks in cocultures with adipose-derived stromal cells**. Scale bar represents 100 µm. **(A,B)** Overlays of fluorescent images showing that BEC and LEC form two separate networks and that upon addition of exogenous vascular endothelial growth factor (VEGF)-C, LEC network becomes increasingly dense. **(C,D)** Merged Z-stacks taken with confocal microscope showing that without exogenous VEGF-C, there is limited proliferation and network formation of LEC, while in the presence of VEGF-C (25 ng/mL), LEC develop an extensive network that is separate from the BEC network. LEC were transduced with GFP and BEC with mCherry.

Considering the observation that LEC form vascular-like networks when cultured with ASC in fibrin hydrogels, we next aimed to determine whether LEC and BEC can form separate networks and tube-like structures when cocultured together in fibrin matrices. Since the concentration of 50 ng/mL of VEGF-C did not result in significantly denser networks compared to 25 ng/mL (Figure 3B), LEC were further cultured in fibrin gels with EGM-2 supplemented with 25 ng/mL of VEGF-C. BEC and LEC were integrated, either as monocultures, as cocultures with ASC or as tricultures containing LEC, BEC, and ASC. The duplicate samples were cultured in EGM-2 medium supplemented with VEGF-C. In the absence of ASC, neither BEC nor LEC could form tube-like structures or networks (Figure 4A), and the cells grew in monolayers. Without VEGF-C, BEC (red) were more prevalent, while with the addition of VEGF-C, the LEC (green) and BEC formed clearly separated clusters of cells. Both LEC and BEC formed networks in the presence of ASC, whereas LEC network formation was more prominent with the addition of exogenous VEGF-C. While the mean numbers of LEC tubules and junctions per field in the absence of VEGF-C were 87 (±6.417) and 31 (±5.266), respectively, with the addition of VEGF-C, the mean numbers of tubules and junctions increased to 450 (±25.94) and 240 (±15.84), respectively. Without VEGF-C, the mean number of tubules of BEC was 452 (±46.46) and 260 (±33.35), respectively, and of junctions was 483 (±52.40) and 241 (±29.34), respectively (Figure 4B). LEC and BEC networks could also be distinguished by their morphology: BEC formed thinner, more uniform structures in comparison to the wider and more variable LEC structures (Figures 4A and 5A,B). This observation was also confirmed by confocal microscopy, where BEC and LEC networks could be clearly distinguished in the presence of VEGF-C, while the LEC network was close to absent when grown in medium lacking VEGF-C (Figures 5C,D).

### Direct Contact between Endothelial Cells and ASC Is Necessary for the Formation of both Lymphatic and Blood Vascular Network-Like Structures

**Figure 6:**
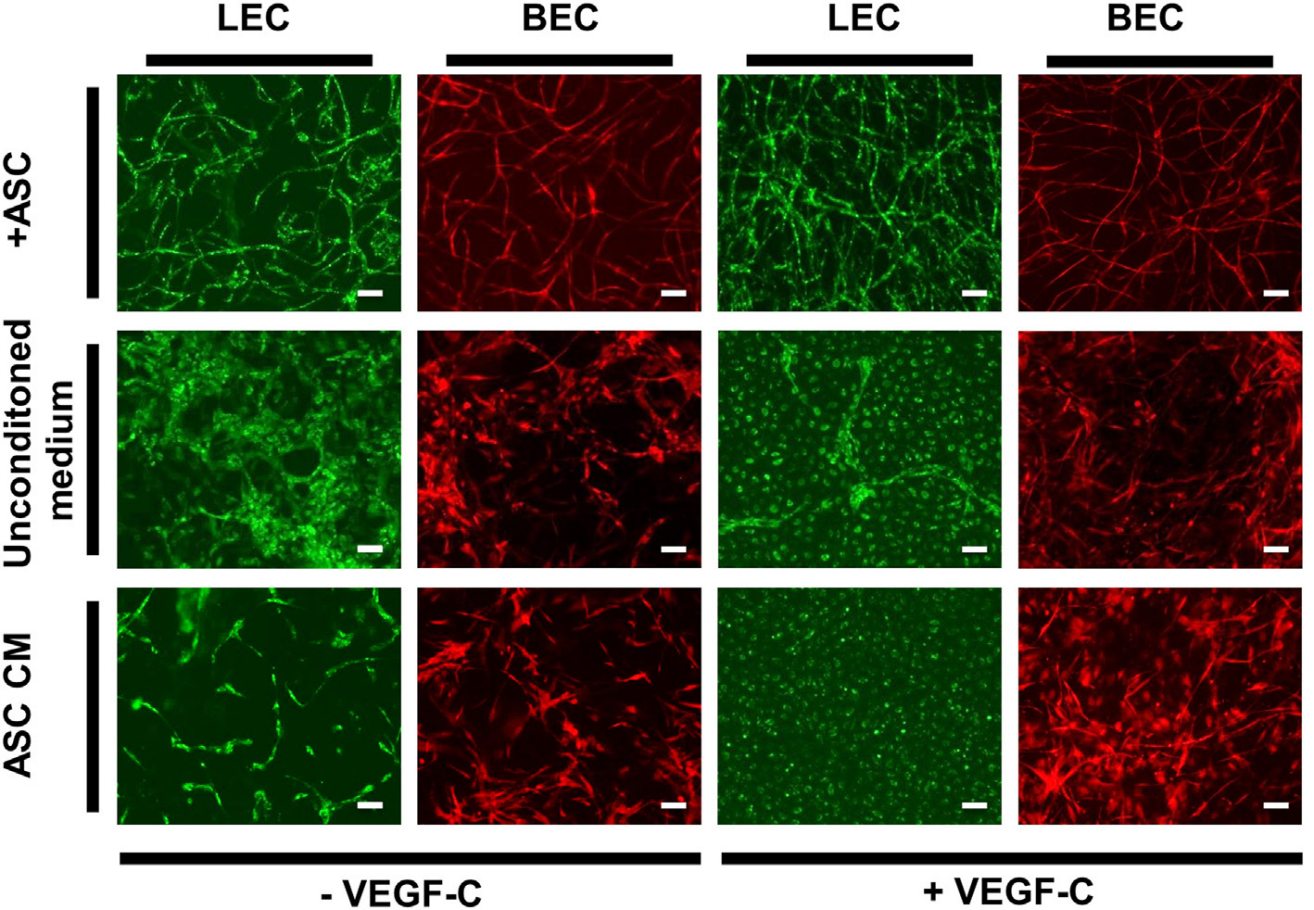
**The effect of ASC-conditioned medium (ASC-CM) on the network formation of lymphatic endothelial cells (LEC) (green) and blood vascular endothelial cells (BEC) (red)**. Scale bar indicates 100 µm. The first row shows successful network formation of both LEC and BEC when cultured with adipose-derived stromal cells (ASC) in EGM-2 medium. The LEC network formation is enhanced upon the addition of vascular endothelial growth factor (VEGF)-C. In the middle row, LEC and BEC were cultured in monocultures in EGM-2 medium, with or without added VEGF-C, which resulted in no network formation of either cell types. In the bottom row, the cells were cultured in the ASC-CM, which, similarly to unconditioned EGM-2, resulted in no network formation of LEC or BEC. Images were taken at day 14 of culture. LEC were transduced with mCherry and BEC with GFP. Images are representative of two experiments with two HDMEC donors.

Adipose-derived stromal cells have been shown previously to support vessel formation by endothelial cells *in vitro* and *in vivo* (Verseijden et al., 2010b; Rohringer et al., 2014; Holnthoner et al., 2015). Additionally, ASC-CM was shown to have a beneficial effect on the migration, proliferation, and network formation of endothelial cells (Merfeld-Clauss et al., 2010; Verseijden et al., 2010b; Rohringer et al., 2014). However, direct cell–cell contact was shown to be essential for full network and tube formation (Rohringer et al., 2014). To examine whether ASC-CM had a similar effect on LEC and BEC network formation as co-culturing, these cells with ASC, LEC, and BEC were embedded in fibrin hydrogels and incubated in ASC-CM. The medium was generated by culturing ASC in EGM-2 for 48 h prior to applying the medium onto LEC and BEC. The cells were also cultured in the presence of ASC to control for the network-forming capacity of BEC and LEC. LEC were grown with the addition of VEGF-C (25 ng/mL), as this was shown to lead to network formation in coculture with ASC. When cultured with ASC, both LEC and BEC showed network formation by day 14 of culture (Figure 6). LEC showed more extensive network formation upon the addition of VEGF-C. There was, again, no effect of VEGF-C on the network formation of BEC. When cultured without ASC in EGM-2 medium, neither LEC nor BEC displayed network formation. With the addition of VEGF-C, LEC proliferated and formed a monolayer. BEC started forming some elongated structures, but no network formation could be observed. When the cells were cultured in ASC-CM, there was again no network formation, and the cells grew similarly to their respective counterparts in unconditioned EGM-2 medium. While BEC formed some elongated structures, they were not able to form a network by day 14. LEC again proliferated in the presence of VEGF-C and grew in a monolayer, while without VEGF-C, the cells started to elongate but could not form a network. Since we did not observe full network formation in the conditions without ASC, these networks could not be quantified and further subjected to statistical analysis.

## Discussion

Integration of both blood and lymphatic vasculature into tissue-engineered constructs can be crucial for the long-term viability and survival of such constructs *in vivo*. In this study, separate blood and lymphatic microvascular structures were successfully created in a 3D *in vitro* model using BEC and LEC derived from human dermis. We could follow the network development over time by fluorescently labeling the two cell populations, which represents a novel approach in studying lymphangiogenesis and vasculogenesis *in vitro*.

Since the human dermal microvascular fraction consists of both BEC and LEC, the two cell populations were firstly isolated separately, before being used in further cocultures. LEC can be distinguished from BEC by the presence of LEC-specific markers such as podoplanin, LYVE-1, prospero homeobox (Prox)-1, and VEGFR3 (Kaipainen et al., 1995; Banerji et al., 1999; Breiteneder-Geleff et al., 1999; Wigle et al., 2002; Cueni and Detmar, 2006). Podoplanin was chosen as a suitable lymphatic marker, as it has been used previously to isolate LEC from a mixed population containing LEC and BEC (Kriehuber et al., 2001; Makinen et al., 2001) due to its almost exclusive expression on lymphatic endothelium (Carreira et al., 2001; Florez-Vargas et al., 2008) and preferential staining of the smaller diameter LEC vessels (Galambos and Nodit, 2005). Immunomagnetic separation of HDMEC based on this marker resulted in a >96% pure podoplanin-negative and -positive populations (Figures 1B,C), where the positive population was expressed LYVE-1, which was not present in the podoplanin-negative BEC population (Figures 1B,C). Although VEGFR3 is a lymphatic endothelium-specific marker, our observations of a VEGFR3-positive BEC population is in accordance with previous data by Kriehuber et al. (2001) who also showed the presence of VEGFR3 on some BEC, albeit to a lesser extent than shown here. The purity of the populations is of great importance for showing the separate network formations of BEC and LEC.

Lymphatic endothelial cells and BEC populations were transduced with fluorescent proteins and cultured together with ASC for a period of 4 weeks to follow the progress of network formation over time. ASC have been shown previously to support vasculogenesis by secretion of pro-vasculogenic factors and direct cell–cell contact (Verseijden et al., 2010b; Rohringer et al., 2014; Holnthoner et al., 2015; Merfeld-Clauss et al., 2015). In addition, ASC support the migration, proliferation, and tube formation of LEC, again through direct contact and the secretion of growth factors, such as VEGF-A, VEGF-D, fibroblast growth factor-2, hepatocyte growth factor, and angiopoietin-1 (Hsiao et al., 2012; Takeda et al., 2015; Strassburg et al., 2016). Since there is controversy in literature regarding the secretion of VEGF-C by ASC in cocultures with endothelial cells (Rohringer et al., 2014; Takeda et al., 2015; Strassburg et al., 2016), the cocultures of LEC, BEC, and ASC were supplemented with 25 ng/mL VEGF-C, which had been shown previously to support LEC proliferation and network formation (Podgrabinska et al., 2002; Boardman and Swartz, 2003; Marino et al., 2014; Gibot et al., 2016). Our results support these observations. Here, we show that LEC and BEC form elongated structures in the presence of ASC and VEGF-C even in the 2D coculture, and these structures were observed to be separate from each other. In addition, the distinction between LEC and BEC structures could be found in their morphology, as LEC formed structures of variable width, while BEC joined into thin, more uniform projections (Figure 2). Based on these observations, we proceeded to integrate LEC, BEC, and ASC into fibrin matrices to observe the network formation in 3D. Fibrin was chosen as a natural product of the blood coagulation cascade (Davie et al., 1991) and as suitable scaffold material as it has been described previously to facilitate successful vasculogenesis (Rohringer et al., 2014; Holnthoner et al., 2015). Without ASC, both LEC and BEC formed a monolayer and did not arrange even into primitive tube-like structures. Coculturing of both LEC and BEC with ASC had a positive effect on the network formation, while this effect was more apparent for BEC than for LEC when grown in EGM-2 medium without exogenous VEGF-C. VEGF-C significantly enhanced the network formation of LEC in cocultures with ASC, but not of BEC, indicating that VEGF-C is essential for the network formation of LEC and that, even if secreted by ASC, the amounts are not sufficient to promote network formation of LEC. Kazenwadel et al. (2012) showed that VEGF-C promotes lymphatic vessel elongation in studies of embryonic mouse LEC, but that, in cooperation with FGF-2, the vessels also grow in diameter. Although we did not measure the vessel diameter, the fluorescent images of LEC grown in different VEGF-C concentrations suggest there are some structural differences between low and high VEGF-C concentrations. The seemingly widest structures, resembling the ones shown by Kazenwadel et al. (2012), are present mostly under the conditions lacking VEGF-C, or at 10 ng/mL. Such differences are not observable in BEC networks, which form similarly dense networks at all concentrations of VEGF-C (data not shown). Most importantly, the two separate endothelial cell populations clearly form two separate networks, lacking a “mosaic” phenotype between LEC and BEC. These observations, together with our flow cytometry data, which support the purity of fluorescent BEC and LEC populations, demonstrates that two separate networks can be formed from the same dermal endothelial cell source. Kriehuber et al. (2001) also showed separate capillary tube formation by LEC and BEC when cultured together on Matrigel. Marino et al. (2014) reported the formation of two separate networks when HDMEC were cultured in fibrin hydrogels by staining the networks against CD31 and the lymphatic marker Prox-1. Their model has also shown successful anastomosis of engineered vasculature with the rat host vasculature *in vivo* (Marino et al., 2014). Helm et al. cocultured BEC and LEC in a 3D matrix consisting of collagen and fibrin with covalently bound VEGF. They, furthermore, applied interstitial flow in order to enhance capillary formation (Helm et al., 2007). The positive effect of interstitial fluid flow on LEC migration was also investigated on an *in vivo* mouse tail model (Boardman and Swartz, 2003) and *in vitro* by using a multichamber fluidic device (Ng et al., 2004). Strassburg et al. cocultured ASC and LEC *in vitro* in Matrigel to achieve tube formation. However, no fully connected lymphatic network was observed (Strassburg et al., 2016). Matrigel is a not well-defined matrix derived from Engelbreth-Holm-Swarm tumors in mice (Hughes et al., 2010). Therefore, non-tumor-derived autologous scaffolds, such as fibrin, are a more feasible and adequate matrix for pre-vascularization strategies. Considering the recent developments in engineering lymphatic microcapillaries, the data presented here build upon the current knowledge of blood and lymphatic microvasculature formation and offer a valuable 3D model that can be followed during a period of 4 weeks.

Finally, we aimed to investigate whether direct contact between ASC and LEC or BEC was necessary for the development of vascular networks, since ASC have been shown previously to support the vessel formation of endothelial cells (Verseijden et al., 2010b; Rohringer et al., 2014; Holnthoner et al., 2015). Additionally, ASC-CM was shown to have a beneficial effect on endothelial cell migration, proliferation, and network formation (Merfeld-Clauss et al., 2010; Verseijden et al., 2010b; Rohringer et al., 2014). Since lymphatic capillaries are not surrounded by mural cells (Swartz and Skobe, 2001), we investigated whether LEC could develop into a fully established lymphatic capillary network in the presence of ASC-CM. In line with previous findings (Verseijden et al., 2010b; Rohringer et al., 2014), we did not observe network formation when LEC or BEC were cultured in ASC-CM, suggesting that cell–cell interaction or even direct contact is necessary for the development of both lymphatic and blood microcapillaries. It is, therefore, possible that while the development of lymphatic capillaries depends on direct contact with supporting cells, LEC, once developed, do not require support from pericytes for maintenance of the vessels.

Pre-vascularization of tissue-engineered constructs requires autologous sources of endothelial cells. Outgrowth endothelial cells can be isolated from peripheral blood (Fuchs et al., 2009) and have shown network-forming capacities in cocultures with ASC (Fuchs et al., 2009; Medina et al., 2010; Holnthoner et al., 2015). LEC were also suggested to be present in peripheral blood (Szöke et al., 2015) and have also been obtained previously from induced pluripotent stem cells (Lee et al., 2015). Interestingly, preliminary data from our group showed that combinations of LEC derived from human lymph nodes in triculture with ASC and HUVEC formed separate vascular networks, too (data not shown). One limitation of our study is the use of HDMEC as a source for both LEC and BEC, which is not a clinically applicable source of these cells. The use of potentially autologous cells in our model would represent an important step toward pre-vascularization of tissue-engineered constructs. Moreover, the endothelium is a highly heterogeneous organ. In this study, we used ECs from human foreskin, which represent a “golden standard” for microvascular ECs. However, ECs from other tissues possibly lead to different results, thereby limiting the conclusions of our data. Furthermore, it is difficult to assess the presence of the vascular lumen in the networks in our experimental setting. However, we have shown previously the presence of perfusable, lumen-containing structures in networks of outgrowth endothelial cells cocultured with ASC in fibrin hydrogels *in vivo* (Pill et al., 2015). While recent studies report the culture of the networks for a period of 24 h up to 10 days (Podgrabinska et al., 2002; Ng et al., 2004; Helm et al., 2007; Kazenwadel et al., 2012), we show here that the two separate networks are sustainable over the period of 4 weeks. Furthermore, our data show that network density of endothelial cells in co- and tricultures increases over time. Implantation of lymphatic and blood vascular networks in fibrin hydrogels, e.g., in a nude mouse model will shed more light on the functionality of our model *in vivo*.

Our 3D model contributes to the reemerging field of lymphatic tissue engineering, and it also represents a useful model for studying the pathogenesis of this system. In addition, the scaffold containing both the lymphatic and blood vascular networks could be used to study the influences of various angiogenic and lymphangiogenic drugs on the development and interactions of the two systems *in vitro*. Furthermore, engineering lymphatic microvasculature would be a valuable addition to microphysiological systems used for drug development and diagnostics (Muehleder et al., 2014; Tien, 2014; Hasenberg et al., 2015). Attempts to generate an artificial lymphatic drainage system on a chip have been made; however, the incorporation of LEC in such a system is currently unprecedented (Wong et al., 2013). Importantly, the integration of blood and lymphatic microcapillaries could enhance the survival of the tissues incorporated in the chips and, furthermore, contribute to a better recapitulation of physiological conditions (Muehleder et al., 2014; Tien, 2014). Considering the *in vivo* validation and acquisition of BEC and LEC from a potentially autologous source, our model could serve as a pre-vascularization strategy of both blood and lymphatic systems leading to improved survival and functionality of engineered tissues.

## Author Contributions

LK, MS, and SM performed experiments. KS and EP isolated and characterized cells for this study. TH, HR, UM, and WH contributed to the concept and outline of this study. LK, MS, and WH wrote the manuscript.

## Conflict of Interest Statement

KS, TH, and UM are employees of TissUse GmbH that produces and markets microphysiological systems. The other authors declare no conflict of interest.

## References

[B1] Baldwin J. Antille M. Bonda U. De-Juan-Pardo E. M. Khosrotehrani K. Ivanovski S. (2014). In vitro pre-vascularisation of tissue-engineered constructs A co-culture perspective. Vasc. Cell 6, 1–16. 10.1186/2045-824X-6-13 25071932 PMC4112973

[B2] Banerji S. Ni J. Wang S. X. Clasper S. Su J. Tammi R. (1999). LYVE-1, a new homologue of the CD44 glycoprotein, is a lymph-specific receptor for hyaluronan. J. Cell Biol. 144, 789–801. 10.1083/jcb.144.4.789 10037799 PMC2132933

[B3] Beesley V. Janda M. Eakin E. Obermair A. Battistutta D. (2007). Lymphedema after gynecological cancer treatment: prevalence, correlates, and supportive care needs. Cancer 109, 2607–2614. 10.1002/cncr.22684 17474128

[B4] Boardman K. C. Swartz M. A. (2003). Interstitial flow as a guide for lymphangiogenesis. Circ. Res. 92, 801–808. 10.1161/01.RES.0000065621.69843.49 12623882

[B5] Breiteneder-Geleff S. Soleiman A. Kowalski H. Horvat R. Amann G. Kriehuber E. (1999). Angiosarcomas express mixed endothelial phenotypes of blood and lymphatic capillaries: podoplanin as a specific marker for lymphatic endothelium. Am. J. Pathol. 154, 385–394. 10.1016/S0002-9440(10)65285-6 10027397 PMC1849992

[B6] Carmeliet P. Jain R. K. (2000). Angiogenesis in cancer and other diseases. Nature 407, 249–257. 10.1038/35025220 11001068

[B7] Carreira C. M. Nasser S. M. Di Tomaso E. Padera T. P. Boucher Y. Tomarev S. I. (2001). LYVE-1 is not restricted to the lymph vessels: expression in normal liver blood sinusoids and down-regulation in human liver cancer and cirrhosis. Cancer Res. 61, 8079–8084. 11719431

[B8] Charwat V. Schütze K. Holnthoner W. Lavrentieva A. Gangnus R. Hofbauer P. (2015). Potential and limitations of microscopy and Raman spectroscopy for live-cell analysis of 3D cell cultures. J. Biotechnol. 205, 70–81. 10.1016/j.jbiotec.2015.02.007 25687101

[B9] Cornelius L. A. Nehring L. C. Roby J. D. Parks W. C. Welgus H. G. (1995). Human dermal microvascular endothelial cells produce matrix metalloproteinases in response to angiogenic factors and migration. J. Invest. Dermatol. 105, 170–176. 10.1111/1523-1747.ep12317080 7543547

[B10] Costa-Almeida R. Granja P. L. Soares R. Guerreiro S. G. (2014). Cellular strategies to promote vascularisation in tissue engineering applications. Eur. Cell. Mater. 28, 51–67. 10.22203/eCM.v028a05 25050838

[B11] Cueni L. N. Detmar M. (2006). New insights into the molecular control of the lymphatic vascular system and its role in disease. J. Invest. Dermatol. 126, 2167–2177. 10.1038/sj.jid.5700464 16983326

[B12] Davie E. W. Fujikawa K. Kisiel W. (1991). The coagulation cascade: initiation, maintenance, and regulation. Biochemistry 30, 10363–10370. 10.1021/bi00107a001 1931959

[B13] DiMaio T. A. Wentz B. L. Lagunoff M. (2016). Isolation and characterization of circulating lymphatic endothelial colony forming cells. Exp. Cell Res. 340, 159–169. 10.1016/j.yexcr.2015.11.015 26597759 PMC4903174

[B14] Duttenhoefer F. Lara De Freitas R. Meury T. Loibl M. Benneker L. M. Richards R. G. (2013). 3D scaffolds co-seeded with human endothelial progenitor and mesenchymal stem cells: evidence of prevascularisation within 7 days. Eur. Cell. Mater. 26, 49–65. 10.22203/eCM.v026a04 23986333

[B15] Elbjeirami W. M. West J. L. (2006). Angiogenesis-like activity of endothelial cells co-cultured with VEGF-producing smooth muscle cells. Tissue Eng. 12, 381–390. 10.1089/ten.2006.12.381 16548696

[B16] Florez-Vargas A. Vargas S. O. Debelenko L. V. Perez-Atayde A. R. Archibald T. Kozakewich H. P. (2008). Comparative analysis of D2-40 and LYVE-1 immunostaining in lymphatic malformations. Lymphology 41, 103–110. 19013877

[B17] Frerich B. Lindemann N. Kurtz-Hoffmann J. Oertel K. (2001). In vitro model of a vascular stroma for the engineering of vascularized tissues. Int. J. Oral Maxillofac. Surg. 30, 414–420. 10.1054/ijom.2001.0130 11720044

[B18] Fuchs S. Ghanaati S. Orth C. Barbeck M. Kolbe M. Hofmann A. (2009). Contribution of outgrowth endothelial cells from human peripheral blood on in vivo vascularization of bone tissue engineered constructs based on starch polycaprolactone scaffolds. Biomaterials 30, 526–534. 10.1016/j.biomaterials.2008.09.058 18977026

[B19] Galambos C. Nodit L. (2005). Identification of lymphatic endothelium in pediatric vascular tumors and malformations. Pediatr. Dev. Pathol. 8, 181–189. 10.1007/s10024-004-8104-9 15719202

[B20] Gibot L. Galbraith T. Kloos B. Das S. Lacroix D. A. Auger F. A. (2016). Cell-based approach for 3D reconstruction of lymphatic capillaries in vitro reveals distinct functions of HGF and VEGF-C in lymphangiogenesis. Biomaterials 78, 129–139. 10.1016/j.biomaterials.2015.11.027 26694987

[B21] Grainger S. J. Putnam A. J. (2011). Assessing the permeability of engineered capillary networks in a 3D culture. PLoS ONE 6:e22086. 10.1371/journal.pone.0022086 21760956 PMC3131402

[B22] Hasenberg T. Mühleder S. Dotzler A. Bauer S. Labuda K. Holnthoner W. (2015). Emulating human microcapillaries in a multi-organ-chip platform. J. Biotechnol. 216, 1–10. 10.1016/j.jbiotec.2015.09.038 26435219

[B23] Hayes S. C. Janda M. Cornish B. Battistutta D. Newman B. (2008). Lymphedema after breast cancer: incidence, risk factors, and effect on upper body function. J. Clin. Oncol. 26, 3536–3542. 10.1200/JCO.2007.14.4899 18640935

[B24] Helm C. L. E. Zisch A. Swartz M. A. (2007). Engineered blood and lymphatic capillaries in 3-D VEGF-fibrin-collagen matrices with interstitial flow. Biotechnol. Bioeng. 96, 167–176. 10.1002/bit.21185 17133613

[B25] Hewett P. W. Murray J. C. (1993). Human microvessel endothelial cells: isolation, culture and characterization. In Vitro Cell Dev. Biol. Anim. 29A, 823–830. 10.1007/BF02631356 8167895

[B26] Holnthoner W. Hohenegger K. Husa A. M. Muehleder S. Meinl A. Peterbauer-Scherb A. (2015). Adipose-derived stem cells induce vascular tube formation of outgrowth endothelial cells in a fibrin matrix. J. Tissue Eng. Regen. Med. 9, 127–136. 10.1002/term.1620 23038666

[B27] Hsiao S. T. Asgari A. Lokmic Z. Sinclair R. Dusting G. J. Lim S. Y. (2012). Comparative analysis of paracrine factor expression in human adult mesenchymal stem cells derived from bone marrow, adipose, and dermal tissue. Stem Cells Dev. 21, 2189–2203. 10.1089/scd.2011.0674 22188562 PMC3411362

[B28] Hughes C. S. Postovit L. M. Lajoie G. A. (2010). Matrigel: a complex protein mixture required for optimal growth of cell culture. Proteomics 10, 1886–1890. 10.1002/pmic.200900758 20162561

[B29] Kaipainen A. Korhonen J. Mustonen T. van Hinsbergh V. W. Fang G. H. Dumont D. (1995). Expression of the fms-like tyrosine kinase 4 gene becomes restricted to lymphatic endothelium during development. Proc. Natl. Acad. Sci. U.S.A 92, 3566–3570. 10.1073/pnas.92.8.3566 7724599 PMC42208

[B30] Kazenwadel J. Secker G. A. Betterman K. L. Harvey N. L. (2012). In vitro assays using primary embryonic mouse lymphatic endothelial cells uncover key roles for fgfr1 signalling in lymphangiogenesis. PLoS ONE 7:e40497. 10.1371/journal.pone.0040497 22792354 PMC3391274

[B31] Kriehuber E. Breiteneder-Geleff S. Groeger M. Soleiman A. Schoppmann S. F. Stingl G. (2001). Isolation and characterization of dermal lymphatic and blood endothelial cells reveal stable and functionally specialized cell lineages. J. Exp. Med. 194, 797–808. 10.1084/jem.194.6.797 11560995 PMC2195953

[B32] Kunz-Schughart L. A. Schroeder J. A. Wondrak M. van Rey F. Lehle K. Hofstaedter F. (2006). Potential of fibroblasts to regulate the formation of three-dimensional vessel-like structures from endothelial cells in vitro. Am. J. Physiol. Cell Physiol. 290, C1385–C1398. 10.1152/ajpcell.00248.2005 16601149

[B33] Lee S.-J. Park C. Lee J. Y. Kim S. Kwon P. J. Kim W. (2015). Generation of pure lymphatic endothelial cells from human pluripotent stem cells and their therapeutic effects on wound repair. Sci. Rep. 5, 11019. 10.1038/srep11019 26066093 PMC4464258

[B34] Makinen T. Veikkola T. Mustjoki S. Karpanen T. Catimel B. Nice E. C. E. (2001). Isolated lymphatic endothelial cells transduce growth, survival and migratory signals via the VEGF-C/D receptor VEGFR-3. EMBO J. 20, 4762–4773. 10.1093/emboj/20.17.4762 11532940 PMC125596

[B35] Marino D. Luginbühl J. Scola S. Meuli M. Reichmann E. (2014). Bioengineering dermo-epidermal skin grafts with blood and lymphatic capillaries. Sci. Transl. Med. 6, 221ra14 10.1126/scitranslmed.3006894 24477001

[B36] Medina R. O’Neill C. L. Humphreys M. W. Gardiner T. A. Stitt A. W. (2010). Outgrowth endothelial cells: characterisation and their potential for reversing ischaemic retinopathy. Invest. Ophthalmol. Vis. Sci. 51, 1–35. 10.1167/iovs.09-4951 20554606

[B37] Merfeld-Clauss S. Gollahalli N. March K. L. Traktuev D. O. (2010). Adipose tissue progenitor cells directly interact with endothelial cells to induce vascular network formation. Tissue Eng. Part A 16, 2953–2966. 10.1089/ten.TEA.2009.0635 20486792 PMC2928048

[B38] Merfeld-Clauss S. Lupov I. P. Lu H. March K. L. Traktuev D. O. (2015). Adipose stromal cell contact with endothelial cells results in loss of complementary vasculogenic activity mediated by induction of activin A. Stem Cells 33, 3039–3051. 10.1002/stem.2074 26037810

[B39] Muehleder S. Ovsianikov A. Zipperle J. Redl H. Holnthoner W. (2014). Connections matter: channeled hydrogels to improve vascularization. Front. Bioeng. Biotechnol. 2:52. 10.3389/fbioe.2014.00052 25453032 PMC4231943

[B40] Ng C. P. Helm C. L. E. Swartz M. A. (2004). Interstitial flow differentially stimulates blood and lymphatic endothelial cell morphogenesis in vitro. Microvasc. Res. 68, 258–264. 10.1016/j.mvr.2004.08.002 15501245

[B41] Petrek J. A. Senie R. T. Peters M. Peterrosen P. (2001). Lymphedema in a cohort of breast carcinoma survivors 20 years after diagnosis. Cancer 92, 1368–1377. 10.1002/1097-0142(20010915)92:6<1368::AID-CNCR1459>3.0.CO;2-9 11745212

[B42] Pill K. Hofmann S. Redl H. Holnthoner W. (2015). Vascularization mediated by mesenchymal stem cells from bone marrow and adipose tissue: a comparison. Cell Regen. (Lond.) 4, 8. 10.1186/s13619-015-0025-8 26500761 PMC4619361

[B43] Podgrabinska S. Braun P. Velasco P. Kloos B. Pepper M. S. Skobe M. (2002). Molecular characterization of lymphatic endothelial cells. Proc. Natl. Acad. Sci. U.S.A. 99, 16069–16074. 10.1073/pnas.242401399 12446836 PMC138566

[B44] Rivron N. C. Liu J. J. Rouwkema J. de Boer J. van Blitterswijk C. A. (2008). Engineering vascularised tissues in vitro. Eur. Cell. Mater. 15, 27–40. 10.22203/eCM.v015a03 18288631

[B45] Rohringer S. Hofbauer P. Schneider K. H. Husa A. M. Feichtinger G. Peterbauer-Scherb A. (2014). Mechanisms of vasculogenesis in 3D fibrin matrices mediated by the interaction of adipose-derived stem cells and endothelial cells. Angiogenesis 17, 921–933. 10.1007/s10456-014-9439-0 25086616

[B46] Rouwkema J. Rivron N. C. van Blitterswijk C. A. (2008). Vascularization in tissue engineering. Trends Biotechnol. 26, 434–441. 10.1016/j.tibtech.2008.04.009 18585808

[B47] Schaupper M. Jeltsch M. Rohringer S. Redl H. Holnthoner W. (2016). Lymphatic vessels in regenerative medicine and tissue engineering. Tissue Eng. Part B Rev. 22, 395–407. 10.1089/ten.TEB.2016.0034 27142568

[B48] Schimek K. Busek M. Brincker S. Groth B. Hoffmann S. Lauster R. (2013). Integrating biological vasculature into a multi-organ-chip microsystem. Lab. Chip 13, 3588–3598. 10.1039/c3lc50217a 23743770

[B49] Siow R. C. M. (2012). Culture of human endothelial cells from umbilical veins. Methods Mol. Biol. 806, 265–274. 10.1007/978-1-61779-367-7_18 22057458

[B50] Sorrell J. M. Baber M. A. Caplan A. I. (2007). A self-assembled fibroblast-endothelial cell co-culture system that supports in vitro vasculogenesis by both human umbilical vein endothelial cells and human dermal microvascular endothelial cells. Cells Tissues Organs 186, 157–168. 10.1159/000106670 17657137

[B51] Strassburg S. Torio-Padron N. Finkenzeller G. Frankenschmidt A. Stark G. B. (2016). Adipose-derived stem cells support lymphangiogenic parameters in vitro. J. Cell. Biochem. 117, 2620–2629. 10.1002/jcb.25557 27018208

[B52] Swartz M. A. Skobe M. (2001). Lymphatic function, lymphangiogenesis, and cancer metastasis. Microsc. Res. Tech. 55, 92–99. 10.1002/jemt.1160 11596154

[B53] Swerlick R. A. Garcia-Gonzalez E. Kubota Y. Xu Y. L. Lawley T. J. (1991). Studies of the modulation of MHC antigen and cell adhesion molecule expression on human dermal microvascular endothelial cells. J. Invest. Dermatol. 97, 190–196. 10.1111/1523-1747.ep12479643 1906507

[B54] Szöke K. Reinisch A. Østrup E. Reinholt F. P. Brinchmann J. E. (2015). Autologous cell sources in therapeutic vasculogenesis: in vitro and in vivo comparison of endothelial colony-forming cells from peripheral blood and endothelial cells isolated from adipose tissue. Cytotherapy 18, 242–252. 10.1016/j.jcyt.2015.10.009 26669908

[B55] Takeda K. Sowa Y. Nishino K. Itoh K. Fushiki S. (2015). Adipose-derived stem cells promote proliferation, migration, and tube formation of lymphatic endothelial cells in vitro by secreting lymphangiogenic factors. Ann. Plast. Surg. 74, 728–736. 10.1097/SAP.0000000000000084 24401810

[B56] Tammela T. Alitalo K. (2010). Lymphangiogenesis: molecular mechanisms and future promise. Cell 140, 460–476. 10.1016/j.cell.2010.01.045 20178740

[B57] Tien J. (2014). Microfluidic approaches for engineering vasculature. Curr. Opin. Chem. Eng. 3, 36–41. 10.1016/j.coche.2013.10.006

[B58] Unger R. E. Ghanaati S. Orth C. Sartoris A. Barbeck M. Halstenberg S. (2010). The rapid anastomosis between prevascularized networks on silk fibroin scaffolds generated in vitro with cocultures of human microvascular endothelial and osteoblast cells and the host vasculature. Biomaterials 31, 6959–6967. 10.1016/j.biomaterials.2010.05.057 20619788

[B59] Verseijden F. Posthumus-van Sluijs S. J. Farrell E. Van Neck J. W. Hovius S. E. R. Hofer S. O. P. (2010a). Prevascular structures promote vascularization in engineered human adipose tissue constructs upon implantation. Cell Transplant. 19, 1007–1020. 10.3727/096368910X492571 20350354

[B60] Verseijden F. Posthumus-van Sluijs S. J. Pavljasevic P. Hofer S. O. P. van Osch G. J. V. M. Farrell E. (2010b). Adult human bone marrow- and adipose tissue-derived stromal cells support the formation of prevascular-like structures from endothelial cells in vitro. Tissue Eng. Part A 16, 101–114. 10.1089/ten.TEA.2009.0106 19642855

[B61] Weitman E. Cuzzone D. Mehrara B. J. (2013). Tissue engineering and regeneration of lymphatic structures. Future Oncol. 9, 1365–1374. 10.2217/fon.13.110 23980683 PMC4095806

[B62] Wigle J. T. Harvey N. Detmar M. Lagutina I. Grosveld G. Gunn M. D. (2002). An essential role for Prox1 in the induction of the lymphatic endothelial cell phenotype. EMBO J. 21, 1505–1513. 10.1093/emboj/21.7.1505 11927535 PMC125938

[B63] Wolbank S. Peterbauer A. Fahrner M. Hennerbichler S. van Griensven M. Stadler G. (2007). Dose-dependent immunomodulatory effect of human stem cells from amniotic membrane: a comparison with human mesenchymal stem cells from adipose tissue. Tissue Eng. 13, 1173–1183. 10.1089/ten.2006.0313 17518752

[B64] Wong K. H. K. Truslow J. G. Khankhel A. H. Chan K. L. S. Tien J. (2013). Artificial lymphatic drainage systems for vascularized microfluidic scaffolds. J. Biomed. Mater. Res. A. 101, 2181–2190. 10.1002/jbm.a.34524 23281125 PMC3620968

